# Incidence and Neonatal Risk factors of Short Stature and Growth Hormone treatment in Japanese Preterm Infants Born Small for Gestational Age

**DOI:** 10.1038/s41598-019-48785-y

**Published:** 2019-08-22

**Authors:** Masaaki Matsumoto, Nobuhiko Nagano, Hiroyuki Awano, Shohei Ohyama, Kazumichi Fujioka, Sota Iwatani, Tatsuhiko Urakami, Kazumoto Iijima, Ichiro Morioka

**Affiliations:** 1 Department of Pediatrics, Kobe University Graduate School of Medicine, Kobe, Japan; 2 Department of Pediatrics and Child Health, Nihon University School of Medicine, Tokyo, Japan

**Keywords:** Outcomes research, Risk factors

## Abstract

Incidence and neonatal risk factors for short stature in preterm children born small for gestational age (SGA) have not been fully investigated in Japan. In this prospective study, infants born ≤32 weeks’ gestational age (GA) from 2004–2015 were enrolled and followed for 3 years. Incidence of short children born SGA and short stature treated with growth hormone (GH) were investigated. Neonatal risk factors were analysed using univariate and multivariate analyses. GA cut-off value was determined using receiver operating characteristic (ROC) curve analyses. Of 604 infants born ≤32 weeks’ GA, 76 (13%) were SGA at birth. Twenty-seven infants (36%) developed short stature at age 2 and 14 infants (19%) received GH treatment at age 3. GA, birthweight, birth length, birth head circumference, and chronic lung disease at 36 weeks’ corrected GA were determined as risk factors by univariate analyses (p < 0.01). Multivariate analyses only revealed low GA as an independent risk factor. ROC curve analysis determined a cut-off value of 24 weeks’ GA. Nineteen percent of preterm SGA infants ≤32 weeks’ GA developed short stature treated with GH. A low GA at birth could be an early detection marker for short stature that requires GH treatment in preterm infants born SGA.

## Introduction

Recent advancements in perinatal management have led to an increase in the survival rate of preterm infants with fetal growth restrictions, resulting in infants who are small for gestational age (SGA) at birth. Around 90% of children who are born SGA achieve normal growth by age 2^1,2^. However, approximately 10% of children born SGA do not, leading to children with short stature^3–5^, which may persist through to adulthood^6,7^. To ensure that children with severe short stature achieve their normal growth, they can be treated with growth hormone (GH)^8,9^. Recent studies have revealed that GH treatment not only helps achieve normal growth, but also improves abnormal lipid and amino acid metabolic conditions^10–12^. Treatment with GH for short children born SGA has been explored for almost 40 years^13,14^ and is currently an approved treatment in many countries^8,15,16^. Thus, monitoring for growth and adequate introduction of GH treatment are important issues in the follow-up of children born SGA.

In terms of follow-up for infants with SGA, it is important to know the incidence and neonatal risk factors associated with short stature that needs GH treatment. We previously reported in a Japanese city population-based study that the estimated incidence of short children born SGA that met the criteria for GH treatment was 0.06% and 1.6% in all 3-year-old children and 3-year-old SGA children, respectively^17^. However, due to the study design, early preterm infants (i.e., ≤32 weeks’ GA) were not included sufficiently. The incidence of short stature increases as gestational age (GA) decreases^4,5,17^. Early preterm infants are generally admitted to neonatal intensive care units, and some demonstrate complications due to prematurity. In early preterm SGA infants, neonatal risk factors for the development of short stature and short stature that is severe enough to warrant treatment with GH may exist; however, they have not yet been fully investigated. Thus, this study aimed to reveal the incidence and neonatal risk factors of short stature and short stature treated with GH in early preterm infants born SGA through a multiple hospital-based prospective cohort study.

## Results

### Incidence

**Figure 1:**
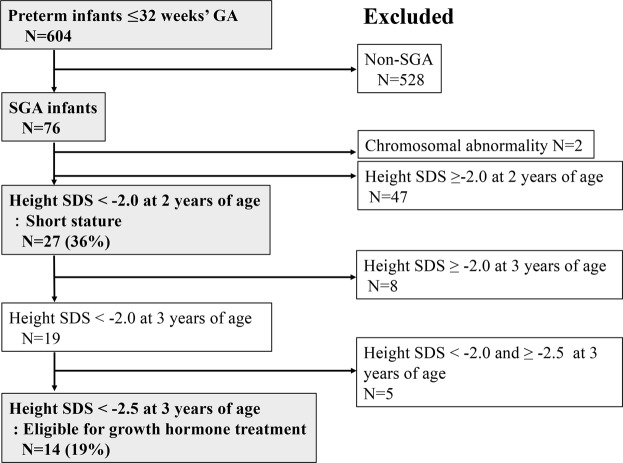
Flowchart of the subject selection and enrolment process. SDS, standard deviation score; SGA, small-for-gestational age.

During the study period in both hospitals combined, a total of 604 infants were born ≤32 weeks’ GA and 76 (13%) were SGA. Two infants were excluded because of chromosomal abnormality. Finally, 74 SGA infants were enrolled in this prospective study. The incidence of short children born SGA and those treated with GH were 36% and 19%, respectively (Fig. 1). Subject selection and enrolment in each hospital are shown in Supplementary Figs 1 and 2. Incidence of short stature and short stature treated with GH between the two hospitals was not significantly different (p = 0.641, p = 0.651, Table 1).

**Table 1:** Comparison of incidence of short stature and short stature treated with GH between the two hospitals.

	Nihon University Itabashi Hospital, n = 41	Kobe University Hospital, n = 33	p-value
Short stature at age 2	14 (34)	13 (39)	0.641
GH treatment at age 3	7 (17)	7 (21)	0.651

Data are shown as number (percentage). GH, growth hormone.

### Associated factors for short stature

Univariate analyses comparing short stature and non-short stature groups revealed that birthweight (BW), birth length (BL), birth head circumference, standard deviation scores (SDSs) of BW, BL, and birth head circumference, GA, Apgar score (1 min, 5 min), duration of artificial ventilation, duration of oxygen therapy, respiratory distress syndrome (RDS), patent ductus arteriosus (PDA), chronic lung disease at 36 weeks’ corrected GA (CLD36), and meconium disease were significantly different between the two groups (Table 2).

**Table 2:** Factors associated with short stature.

	Short stature		p-value
	Yes, n = 27	No, n = 47	
BW, g	568 (314–1174)	998 (476–1426)	<0.001
BW SDS	−3.14 (−4.28–−1.97)	−2.46 (−3.97–−1.39)	<0.001
BL, cm	28.5 (23.5–38.5)	35.0 (26.0–39.0)	<0.001
BL SDS	−2.66 (−4.14–−1.45)	−2.37 (−4.14–−1.30)	0.020
Birth head circumference, cm	22.4 (13.5–27.0)	26.4 (20.0–33.4)	<0.001
Birth head circumference SDS	−1.62 (−2.75–−0.51)	−0.86 (−2.74–+0.34)	<0.001
Male	17 (63)	22 (47)	0.180
GA, weeks	27 (24–32)	30 (26–32)	<0.001
Apgar score, 1 min	4 (1–8)	7 (1–9)	<0.002
Apgar score, 5 min	7 (2–9)	9 (3–10)	<0.001
Use of mechanical artificial ventilation	24 (89)	39 (83)	0.492
Use of oxygen therapy	27 (100)	46 (98)	0.445
Duration of mechanical artificial ventilation, days	53 (0–617)	12 (0–94)	<0.001
Duration of oxygen therapy, days	74 (2–617)	36 (3–119)	<0.001
CLD36	22 (82)	18 (38)	<0.001
Sepsis	2 (7)	3 (6)	0.866
RDS	20 (74)	23 (49)	0.035
PDA	4 (15)	0 (0)	0.007
ROP	5 (19)	4 (9)	0.204
NEC	1 (4)	0 (0)	0.184
Meconium disease	8 (29.6)	5 (11)	0.039
IVH	1 (4)	2 (4)	0.908
PVL	1 (4)	2 (4)	0.908

The values represent those for children with or without short stature. Values are shown as median (range) or number (percentage).

BH, birth height; BW, birth weight; CLD36, chronic lung disease at 36 weeks’ corrected GA; GA, gestational age; IVH, intraventricular haemorrhage; NEC, necrotizing enterocolitis; PDA, patent ductus arteriosus; PVL, periventricular leukomalacia; RDS, respiratory distress syndrome; ROP, retinopathy of prematurity; SDS, standard deviation score.

### Associated factors for GH treatment

Univariate analyses comparing GH treatment and non-GH treatment groups revealed that BW, BL, birth head circumference, SDSs of BW, BL, and birth head circumference, duration of artificial ventilation, duration of oxygen therapy, CLD36, and necrotising enterocolitis (NEC) were significantly different between the two groups (Table 3).

**Table 3:** Factors associated with GH treatment.

	GH treatment		p-value
	Yes, n = 14	No, n = 60	
BW, g	588 (314–1089)	925 (380–1426)	0.002
BW SDS	−3.78 (−4.28–−2.57)	−2.5 (−3.97–−1.39)	<0.001
BL, cm	29.7 (23.5–36.1)	34.0 (25.0–39.0)	0.008
BL SDS	−3.29 (−4.14–−2.15)	−2.39 (−4.14–−1.30)	<0.001
Birth head circumference, cm	23.0 (13.5–27.0)	26.0 (19.0–33.4)	0.003
Birth head circumference SDS	−1.72 (−2.75–−0.75)	−0.96 (−2.74–+0.34)	<0.001
Male	9 (64)	30 (50)	0.335
GA, weeks	28.5 (24–32)	30 (24–32)	0.197
Apgar score, 1 min	4 (1–8)	7 (1–9)	0.116
Apgar score, 5 min	7 (3–9)	8 (2–10)	0.058
Use of mechanical artificial ventilation	12 (86)	51 (85)	0.946
Use of oxygen therapy	14 (100)	59 (98)	0.627
Duration of mechanical artificial ventilation, days	49 (0–179)	19 (0–617)	0.035
Duration of oxygen therapy, days	67 (2–225)	39 (3–617)	0.020
CLD36	12 (86)	28 (47)	0.008
Sepsis	1 (7)	4 (7)	0.949
RDS	8 (57)	35 (58)	0.935
PDA	1 (7)	3 (5)	0.750
ROP	3 (21)	6 (10)	0.239
NEC	1 (7)	0 (0)	0.037
Meconium disease	4 (29)	9 (15)	0.230
IVH	1 (7)	2 (3)	0.515
PVL	0 (0)	3 (5)	0.393

The values represent those for short children with or without GH treatment. Values are shown as median (range) or number (percentage).

BH, birth height; BW, birth weight; CLD36, chronic lung disease at 36 weeks’ corrected GA; GA, gestational age; GH, growth hormone; IVH, intraventricular haemorrhage; NEC, necrotising enterocolitis; PDA, patent ductus arteriosus; PVL, periventricular leukomalacia; RDS, respiratory distress syndrome; ROP, retinopathy of prematurity SDS, standard deviation score.

### Multivariate analyses

Multiple logistic regression analyses using GA, BW, BL, birth head circumference, and CLD36 were performed. Only GA was identified as an independently associated factor for the development of short stature and short stature with GH treatment (odds ratio: 0.45 and 0.14, respectively, Table 4).

**Table 4:** Multivariate logistic regression analyses.

Short stature		
Factors	OR (95% CI)	p-value
GA	0.45 (0.20–0.97)	0.042
BW	1.00 (0.99–1.01)	0.546
BL	1.07 (0.65–1.77)	0.791
Birth head circumference	2.47 (0.90–6.81)	0.080
CLD36	2.55 (0.49–13.39)	0.268
**GH treatment**		
**Factors**	**OR (95% CI)**	**p-value**
GA	0.14 (0.04–0.50)	0.003
BW	1.00 (0.99–1.02)	0.418
BL	1.36 (0.66–2.82)	0.408
Birth head circumference	2.81 (0.80–9.91)	0.109
CLD36	7.44 (0.83–66.70)	0.073

BH, birth height; BW, birth weight; CI, confidence interval; CLD, chronic lung disease; GA, gestational age; GH, growth hormone; OR, odds ratio.

### GA cut-off value for GH treatment

A GA cut-off value of 24 weeks was associated with GH treatment according to the Youden index based on the receiver-operating characteristic (ROC) curve analysis (Table 5).

**Table 5:** GA cut-off value for GH treatment.

GA, weeks	Sensitivity	Specificity	Accuracy	Youden index
**24**	**0.400**	**0.826**	**0.797**	**0.226**
25	0.375	0.833	0.790	0.208
26	0.286	0.833	0.730	0.119
27	0.200	0.815	0.649	0.015
28	0.259	0.851	0.635	0.110
29	0.278	0.895	0.595	0.173
30	0.208	0.846	0.432	0.054
31	0.207	0.875	0.351	0.082
32	0.189	1.000	0.189	0.189

The Youden index is the point farthest from the boundary delineating the area under the curve and represents the [sensitivity + specificity − 1] value^31^.

GA, gestational age; GH, growth hormone.

## Discussion

In this Japanese prospective study, we revealed that the incidences of short stature and short stature treated with GH were as high as 36% and 19% in SGA infants born at ≤32 weeks’ GA, respectively. This incidence rate was high compared to that in term or late-preterm SGA infants^4,17^. We also found that a low GA at birth was an independent risk factor for developing short stature requiring GH treatment.

Maeyama *et al*. have shown that 2.4%, 5.5%, and 6.5% of SGA infants with ≤-2.0 SDS for BW and/or BL developed short stature when born at 39–41, 37–38, and 34–36 weeks’ GA, respectively^4^. Compared with the results of Maeyama *et al*.^4^, our study surprisingly found the incidence in SGA infants ≤32 weeks’ GA to be 15–36 and 5.5 times higher in term and late-preterm infants, respectively. Thus, the incidence has GA dependency. Regarding the incidence of short stature that required GH treatment, Fujita *et al*. reported 15 (1.6%)/956 SGA infants with ≤−2.0 SDS for BW and/or BL, who qualified for GH treatment in a Japanese city population-based study^17^. The study by Fujita *et al*. enrolled mainly term SGA infants (864 of 956, 90%)^17^. In comparison, the incidence was 12 times higher for short stature treated with GH in SGA infants ≤32 weeks’ GA.

In previous Japanese cohort studies^4,5,17^, it was difficult to investigate clinical backgrounds because of insufficient data for preterm infants, especially extremely preterm infants. Therefore, we conducted the current multiple hospital-based prospective study to address this problem. Furthermore, as preterm SGA infants generally receive some treatments in the neonatal intensive care unit due to prematurity, we were concerned whether neonatal factors or complications influenced the development of short stature and short stature that needed GH treatment. In the univariate analysis of our current study on SGA preterm infants, GA, physical stature at birth, and CLD were factors mainly associated with developing short stature and short stature treated with GH. CLD often is associated with a high breathing work load and may affect short stature via a reduced appetite or need for extra calories for growth^18^. Several previous studies have identified factors associated with development of short stature in SGA infants, including GA^5,17^, BW^6^, BL^6,19^, and short height of parents, especially the mother^20^. Our multivariate analyses showed that low GA is an independent risk factor. It is unclear how GA is involved in the development of short stature, but possible mechanisms include gene modifications associated with GH resistance and the intrauterine environment. There is increasing evidence that SGA children who developed short stature had a persistent abnormality in the GH/insulin-like growth factor (IGF)/insulin-like growth factor binding protein (IGFBP) axis^21–24^. The endocrine profile of short children born SGA is characterised by low levels of IGF-1, IGF-2, and IGFBP-3^21,25^. These findings suggest GH resistance in short children born SGA. The correlation between short stature and epigenetic variability in the gene for IGFBP-3, resulting in the reduction of IGFBP-3 expression^26^, has been reported^22^. Preterm infants show altered DNA methylation in the gene encoding IGF-2^27^. The mechanism of short stature in preterm SGA infants with a low GA may associate with IGF-related epigenetic factors.

The current study clarified that a low GA at birth was the highest risk factor for needing GH treatment among preterm SGA infants. Because puberty occurs early in preterm children with low GA^28^, the effective period of GH treatment would be short, and accordingly, the final adult height could be short. If the GH treatment is initiated early in age, the final adult height would be achieved to the corresponding level^29^. Therefore, it is essential to identify short stature and begin treatment as soon as possible in preterm SGA infants.

This study has some limitations. First, we could not collect the parental height of the subjects. Mid-parental heights correlated with adult height in SGA infant^19^. However, the relationship with the height in early childhood is unknown, especially in those below age 3 years. Second, preterm SGA infants consumed a similar number of nutritional calories during the neonatal intensive care unit stay and before weaning, based on the instruction of board-certified neonatologists. However, the received nutrition levels could not be evaluated after weaning. Finally, detailed causes of fetal growth restriction that lead to SGA were not collected; however, in our subjects, causes were abnormalities in the mother, placenta, or umbilical cord, such as pregnancy-induced hypertension and placental dysfunction. Regardless of these limitations, our Japanese prospective study clearly demonstrated that 36% of 74 preterm SGA infants ≤32 weeks’ GA developed short stature and 19% of those were treated with GH. Furthermore, a low GA at birth could be an early detection marker for short stature that requires GH treatment in preterm infants born SGA. Further clinical studies using a large cohort should be performed to confirm the conclusions.

## Methods

### Study design

A multiple hospital-based prospective cohort study for infants who were born at ≤32 weeks’ GA between 2004 and 2014 and followed-up for 3 years was conducted at Kobe University Hospital, Kobe, Japan (infants were enrolled between 2004 and 2012), and Nihon University Itabashi Hospital, Tokyo, Japan (infants were enrolled between 2011 and 2015). This study was approved by the Ethics Committee of Kobe University Graduate School of Medicine (no. 160089) and Nihon University School of Medicine (no. RK-180911-24). Written informed consent was obtained from the parents of all infants. This study was carried out in accordance with the relevant guidelines and regulations.

The following neonatal data were collected: BW, BL, birth head circumference, gender, gestational age, Apgar score (1 min, 5 min), and neonatal complications such as duration of mechanical artificial ventilation, duration of oxygen administration, CLD36, sepsis, RDS, PDA, retinopathy of prematurity, mechanical artificial ventilation, oxygen administration, NEC, meconium disease, intraventricular haemorrhage, and periventricular leukomalacia. Follow-up data collected included height and body weight measured at age 2 and 3 years. Incidences of children with short stature and short stature treated with GH were determined. Neonatal risk factors were then statistically analysed.

### Definition of SGA, short stature, and short stature that requires GH treatment

SGA, short stature, and short stature that requires GH treatment were defined using the following criteria, which were based on sex-specific Japanese standards and guidelines^8^. SGA was defined as both BW and BL < 10th percentile and BW and/or BL < −2.0 SDS. Short stature was defined as SGA and height SDS at age 2 years < −2.0. Short stature with GH treatment was defined as short stature, height SDS at age 3 years < −2.5, and height velocity SDS for 1 year < 0.

### Definitions of neonatal diseases

BW was measured using a digital weight scale for newborns by trained nurses. BL and birth head circumference were measured with a ruler tape. RDS was defined as requirement of artificial surfactant replacement therapy. Sepsis was determined in those with elevated serum C-reactive protein (≥0.5 mg/dl), bacterial pathogen detected from culture, and requiring antibiotic therapy. PDA was determined in those requiring indomethacin administration or surgical ligation. Retinopathy of prematurity was determined in those requiring photocoagulation therapy. NEC was determined in those requiring surgical intervention and confirmed pathological finding. Meconium disease was diagnosed in those treated with administration of a contrast agent into the digestive tract and/or surgical intervention for colostomy. Intracranial haemorrhage and periventricular leukomalacia were diagnosed by brain ultrasonography and magnetic resonance imaging. CLD was diagnosed in those who exhibited evidence of abnormalities on a chest X-ray and were dependent on oxygen therapy at 36 weeks’ corrected GA.

### Physical data at follow-up

Physical data at follow-up were collected at age 2 and 3 years. Height was measured with a digital height meter and body weight was measured with a digital scale.

### Calculation of SDS

SDSs for BW, BL, birth head circumference, and height were calculated using nordiFIT® (Novo Nordisk Pharma, Tokyo, Japan). This can calculate SDSs for BW, BL, and birth head circumference by GA, and height during early childhood, according to sex-specific standards based on the Japanese population^5,17,30^.

### Statistical analyses

The aforementioned neonatal factors associated with short stature and short stature that required GH treatment were analysed using the Wilcoxon signed-rank test or Fisher exact test as univariate analyses, and multiple logistic regression analyses. A cut-off value for GA associated with GH treatment was determined using the maximum Youden index in the ROC curve. The Youden index is the point farthest from the boundary delineating the area under the curve and represents the [sensitivity + specificity − 1] value^31^. Statistical analyses were performed using JMP version 14 (SAS Institute Inc., Tokyo, Japan). A p-value < 0.05 was considered statistically significant.

## Supplementary information

Supplementary Figures 1 and 2

## Data Availability

The data used in this report are available from the corresponding author on reasonable request.

## References

[1] Ong KK, Ahmed ML, Emmett PM, Preece MA, Dunger DB (2000). Association between postnatal catch-up growth and obesity in childhood: prospective cohort study. BMJ..

[2] Albertsson-Wikland K, Karlberg J (1997). Postnatal growth of children born small for gestational age. Acta Paediatr. Suppl..

[3] Itabashi K (2007). Longitudinal follow-up of height up to five years of age in infants born preterm small for gestational age; comparison to full-term small for gestational age infants. Early Hum. Dev..

[4] Maeyama K (2016). Gestational age-dependency of height and body mass index trajectories during the first 3 years in Japanese small-for-gestational age children. Sci. Rep..

[5] Nagasaka M (2015). Incidence of short stature at 3 years of age in late preterm infants: a population-based study. Arch. Dis. Child..

[6] Hokken-Koelega AC (1995). Children born small for gestational age: do they catch up?. Pediatr. Res..

[7] Karlberg J, Albertsson-Wikland K (1995). Growth in full-term small-for-gestational-age infants: from birth to final height. Pediatr. Res..

[8] Tanaka T (2015). Onset of puberty and near adult height in short children born small for gestational age and treated with GH: Interim analysis of up to 10 years of treatment in Japan. Clin. Pediatr. Endocrinol..

[9] Boguszewski MC, Lindberg A, Wollmann HA (2014). Three-year growth response to growth hormone treatment in very young children born small for gestational age-data from KIGS. J. Clin. Endocrinol. Metab..

[10] Kappelgaard AM, Kiyomi F, Horikawa R, Yokoya S, Tanaka T (2014). The impact of long-term growth hormone treatment on metabolic parameters in Japanese patients with short stature born small for gestational age. Horm. Res. Paediatr..

[11] Hirayama S (2017). Growth hormone activates hepatic and cerebral cholesterol metabolism in small-for-gestational age children without catch-up growth. J. Clin. Lipidol..

[12] Nagasaka H (2018). Blood asymmetric dimethylarginine and nitrite/nitrate concentrations in short-stature children born small for gestational age with and without growth hormone therapy. J. Int. Med. Res..

[13] Tanner JM, Ham TJ (1969). Low birthweight dwarfism with asymmetry (Silver’s syndrome): treatment with human growth hormone. Arch. Dis. Child..

[14] Lee PA, Blizzard RM, Cheek DB, Holt AB (1974). Growth and body composition in intrauterine growth retardation (Iugr) before and during human growth-hormone administration. Metabolism..

[15] Lee PA, Chernausek SD, Hokken-Koelega AC, Czernichow P (2003). International Small for Gestational Age Advisory Board consensus development conference statement: management of short children born small for gestational age, April 24-October 1, 2001. Pediatrics..

[16] Clayton PE (2007). Management of the child born small for gestational age through to adulthood: a consensus statement of the International Societies of Pediatric Endocrinology and the Growth Hormone Research Society. J. Clin. Endocrinol. Metab..

[17] Fujita K (2016). Prevalence of small for gestational age (SGA) and short stature in children born SGA who qualify for growth hormone treatment at 3 years of age: Population-based study. Pediatr. Int..

[18] Francis DK, Smith J, Saljuqi T, Watling RM (2015). Oral protein calorie supplementation for children with chronic disease. Cochrane Database Syst. Rev..

[19] Leger J, Limoni C, Collin D, Czernichow P (1998). Prediction factors in the determination of final height in subjects born small for gestational age. Pediatr. Res..

[20] Edouard T (2004). Extreme short stature after intrauterine growth retardation: factors associated with lack of catch-up growth. Horm. Res..

[21] de Waal WJ, Hokken-Koelega AC, Stijnen T, de Muinck Keizer-Schrama SM, Drop SL (1994). Endogenous and stimulated GH secretion, urinary GH excretion, and plasma IGF-I and IGF-II levels in prepubertal children with short stature after intrauterine growth retardation. The Dutch Working Group on Growth Hormone. Clin. Endocrinol. (Oxf)..

[22] van der Kaay DC (2009). Genetic and epigenetic variability in the gene for IGFBP-3 (IGFBP3): correlation with serum IGFBP-3 levels and growth in short children born small for gestational age. Growth Horm. IGF Res..

[23] Albertsson-Wikland K, Boguszewski M, Karlberg J (1998). Children born small-for-gestational age: postnatal growth and hormonal status. Horm. Res..

[24] de Zegher F (2000). Growth hormone treatment of short children born small for gestational age: growth responses with continuous and discontinuous regimens over 6 years. J. Clin. Endocrinol. Metab..

[25] Leger J, Noel M, Limal JM, Czernichow P (1996). Growth factors and intrauterine growth retardation. II. Serum growth hormone, insulin-like growth factor (IGF) I, and IGF-binding protein 3 levels in children with intrauterine growth retardation compared with normal control subjects: prospective study from birth to two years of age. Study Group of IUGR. Pediatr. Res..

[26] Hanafusa T (2002). Reduced expression of insulin-like growth factor binding protein-3 and its promoter hypermethylation in human hepatocellular carcinoma. Cancer Lett..

[27] Piyasena C (2016). Dynamic changes in DNA methylation occur during the first year of life in preterm infants. Front. Endocrinol. (Lausanne)..

[28] Hui LL, Lam HS, Leung GM, Schooling CM (2017). Duration of puberty in preterm girls. Am. J. Hum. Biol..

[29] Dahlgren J, Wikland KA (2005). Final height in short children born small for gestational age treated with growth hormone. Pediatr. Res..

[30] Itabashi K, Miura F, Uehara R, Nakamura Y (2014). New Japanese neonatal anthropometric charts for gestational age at birth. Pediatr. Int..

[31] Fluss R, Faraggi D, Reiser B (2005). Estimation of the Youden Index and its associated cutoff point. Biom. J..

