# Supplementary material for: Incidence and Neonatal Risk factors of Short Stature and Growth Hormone treatment in Japanese Preterm Infants Born Small for Gestational Age
Source: Sci Rep. 2019 Aug 22;9:12238. doi: 10.1038/s41598-019-48785-y (PMC6706397; doi:10.1038/s41598-019-48785-y)
Supplement: Supplementary file 1 — Supplementary Figures 1 and 2 [file 41598_2019_48785_MOESM1_ESM.pdf]

Incidence and Neonatal Risk factors of Short Stature and growth hormone treatment in Japanese Preterm Infants Born Small for Gestational Age

Masaaki Matsumoto[1]*, Nobuhiko Nagano[2]*, Hiroyuki Awano[1], Shohei Ohyama, [1], Kazumichi Fujioka[1], Sota Iwatani[1], Tatsuhiko Urakami[2], Kazumoto Iijima[1], Ichiro Morioka

[1]Department of Pediatrics, Kobe University Graduate School of Medicine, Kobe, Japan
[2]Department of Pediatrics and Child Health, Nihon University School of Medicine, Tokyo, Japan

* These authors contributed equally.

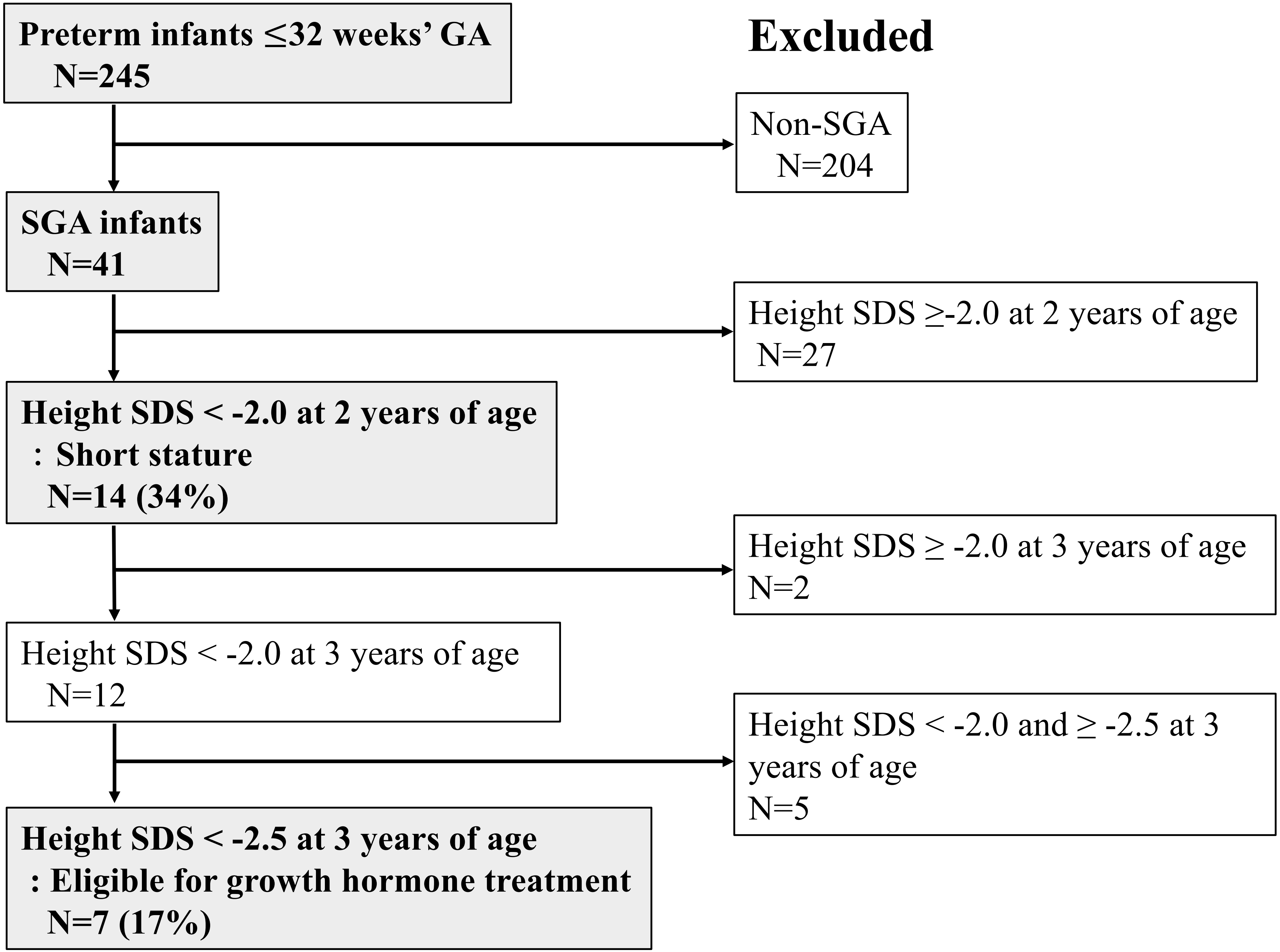

Supplementary Figure 1. Flowchart of the subject selection and enrolment process at Nihon University Itabashi Hospital. SDS, standard deviation score; SGA, small-for-gestational age.

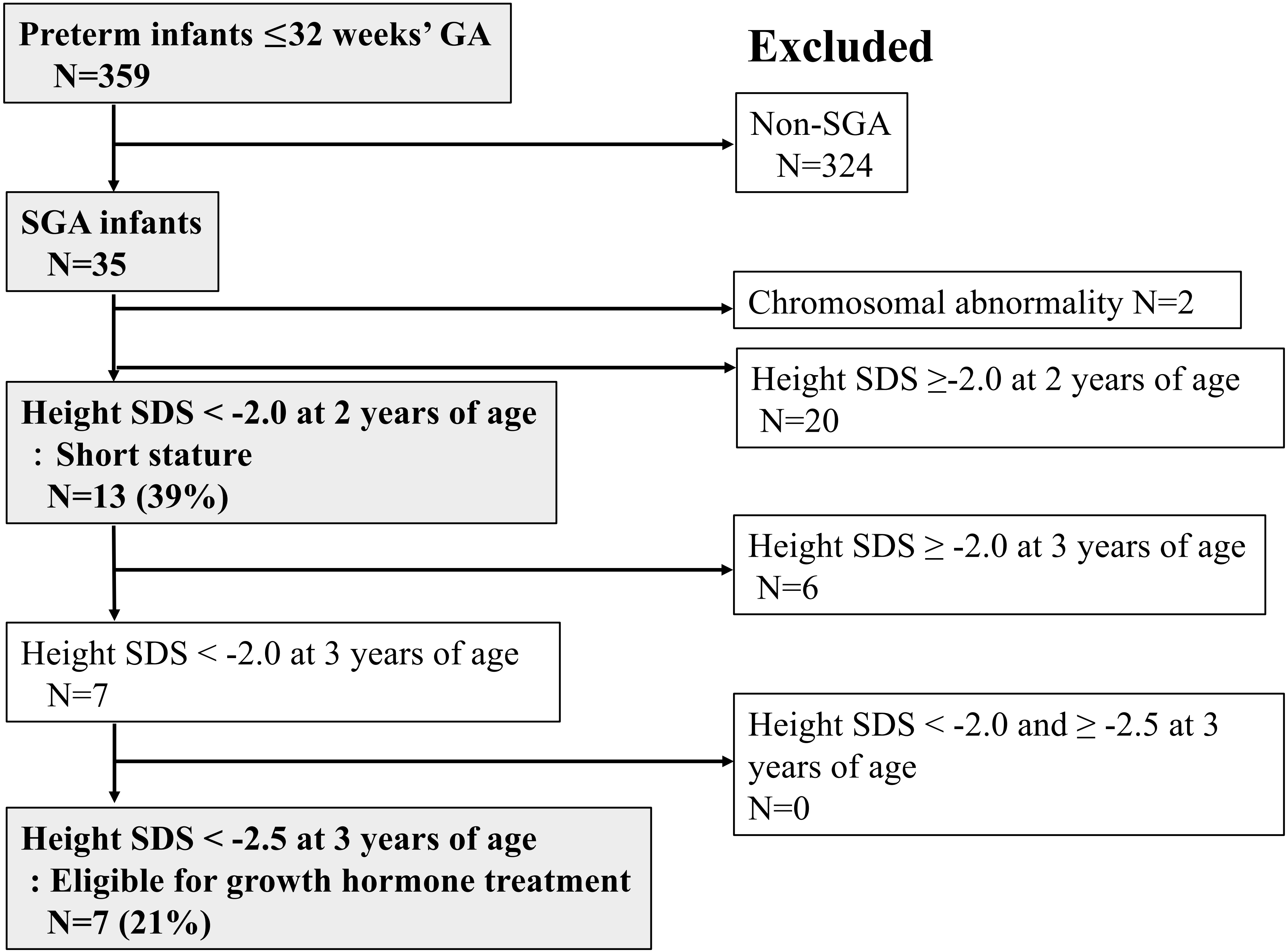

Supplementary Figure 2. Flowchart of the subject selection and enrolment process at Kobe University Hospital. SDS, standard deviation score; SGA, small-for-gestational age.
